# A randomised study and an extension study of brexpiprazole in patients with borderline personality disorder

**DOI:** 10.1017/neu.2024.31

**Published:** 2024-11-19

**Authors:** Brian Rothman, Claudette Brewer, Denise Chang, Mary Hobart, Nanco Hefting, Robert D. McQuade, Jon E. Grant

**Affiliations:** 1 Otsuka Pharmaceutical Development & Commercialization Inc., Princeton, NJ, USA; 2 H. Lundbeck A/S, Valby, Denmark; 3 Department of Psychiatry & Behavioral Neuroscience, Pritzker School of Medicine, University of Chicago, Chicago, IL, USA

**Keywords:** Antipsychotic, borderline personality disorder, clinical trial, double-blind method, pharmacotherapy

## Abstract

**Objective:**

No drugs are currently approved for the treatment of borderline personality disorder (BPD). These studies (a randomised study and its open-label extension) aimed to evaluate the efficacy, safety and tolerability of brexpiprazole for the treatment of BPD.

**Methods:**

The Phase 2, multicentre, randomised, double-blind, placebo-controlled, parallel-group study enrolled adult outpatients with BPD. After a 1-week placebo run-in, patients were randomised 1:1 to brexpiprazole 2–3 mg/day (flexible dose) or placebo for 11 weeks. The primary endpoint was change in Zanarini Rating Scale for BPD total score from randomisation (Week 1) to Week 10 (timing of randomisation and endpoint blinded to investigators and patients). The Phase 2/3, multicentre, open-label extension study enrolled patients who completed the randomised study; all patients received brexpiprazole 2–3 mg/day (flexible dose) for 12 weeks. Safety assessments included treatment-emergent adverse events (TEAEs).

**Results:**

Brexpiprazole was not statistically significantly different from placebo on the primary endpoint of the randomised study (*N* = 324 randomised; *N* = 110 analysed per treatment group; least squares mean difference −1.02; 95% confidence limits −2.75, 0.70; *p* = 0.24). Numerical efficacy advantages for brexpiprazole were observed at other time points. The most common TEAE in the randomised study was akathisia (brexpiprazole, 14.0%; placebo, 1.2%); data from the open-label study (*N* = 199 analysed) suggested that TEAEs were transient.

**Conclusion:**

The primary endpoint of the randomised study was not met. Further research on brexpiprazole in BPD is warranted based on possible efficacy signals at other time points and its safety profile.

ClinicalTrials.gov identifiers: NCT04100096, NCT04186403. Funding: Otsuka, Lundbeck.

## Significant outcomes

- The primary endpoint of the randomised study was not met.
- Numerical efficacy advantages were observed at other time points, and further research is needed to determine the specific value of brexpiprazole in the difficult-to-treat population of patients with borderline personality disorder.
- Brexpiprazole had a similar safety profile in borderline personality disorder to that established in schizophrenia and major depressive disorder.

## Limitations

- The generalisability of the patient sample in these studies is limited by the exclusion of certain populations, including adolescents and patients receiving psychotherapy for BPD symptoms.

## Introduction

Borderline personality disorder (BPD) is characterised by emotional instability, impulsivity, unstable identity and disturbed interpersonal relations, which can result in aggressive outbursts, suicidal ideation and repeated self-injury (Bohus *et al*., 2021). The majority of patients with BPD have comorbidities, most commonly mood, anxiety, and substance use disorders (Tomko *et al*., 2014; Skoglund *et al*., 2021), with overlapping symptoms that can complicate BPD diagnosis (Beatson and Rao, 2013; Ford and Courtois, 2014; Baryshnikov *et al*., 2015). BPD is associated with persistent social and occupational dysfunction, despite high rates of remission at 10 years’ follow-up (Gunderson *et al*., 2011; Alvarez-Tomás *et al*., 2017). The recommended primary treatment for BPD is outpatient psychotherapy (Simonsen *et al*., 2019). No drugs are currently approved for BPD; however, pharmacotherapies—including atypical antipsychotics—are widely used off-label to target specific symptoms, and to treat comorbidities (Bridler *et al*., 2015; Stoffers-Winterling *et al*., 2020; Gartlehner *et al*., 2021). Many atypical antipsychotics are associated with clinically significant side effects (NCCMH, 2009; Huhn *et al*., 2019), indicating the need for a well-tolerated treatment option for BPD.

Brexpiprazole acts as a partial agonist at serotonin 5-HT_1A_ and dopamine D_2_ receptors, and an antagonist at serotonin 5-HT_2A_ and noradrenaline α_1B_/α_2C_ receptors, all with subnanomolar affinity (Maeda *et al*., 2014). In clinical trials and pooled analyses, brexpiprazole has demonstrated efficacy and safety for the treatment of schizophrenia (Correll *et al*., 2015; Kane *et al*., 2015; Marder *et al*., 2017), as an adjunctive treatment in major depressive disorder (MDD) (Thase *et al*., 2015a, 2015b; Hobart *et al*., 2018a, 2018b; Thase *et al*., 2019), and for the treatment of agitation associated with dementia due to Alzheimer’s disease (Grossberg *et al*., 2020; Lee *et al*., 2023).

A single-centre clinical trial (*N* = 80 randomised) suggested that brexpiprazole may show efficacy in BPD (Grant *et al*., 2021); this finding needs to be replicated in a well-powered study. The present article describes two studies of brexpiprazole in BPD: a Phase 2 multicentre randomised controlled study and a Phase 2/3 open-label extension study. The aim of the randomised study was to evaluate the efficacy, safety and tolerability of brexpiprazole for the treatment of BPD. The aim of the extension study was to further assess the safety and tolerability of brexpiprazole in patients with BPD (efficacy was an exploratory objective).

## Methods

### Participants and study design

#### Randomised controlled study

This was a multicentre, randomised, double-blind, placebo-controlled, parallel-group study of brexpiprazole in adult patients with BPD (ClinicalTrials.gov identifier: NCT04100096). Patients were enrolled by investigators at 62 sites in the United States and Europe (Spain, Ukraine), and assessed for eligibility during a screening period of up to 21 days. Key inclusion criteria were outpatient status; age 18–65 years; diagnosis of BPD (Diagnostic and Statistical Manual of Mental Disorders, Fifth edition [DSM-5] criteria) confirmed by the Structured Clinical Interview for DSM-5 Personality Disorders (First *et al.,* 2016); a Zanarini Rating Scale for BPD (ZAN-BPD) total score ≥12 (Zanarini, 2003); a score ≥2 on two or more of the following ZAN-BPD items: inappropriate anger, paranoid ideation, affective instability and impulsivity; and requiring treatment with a medication for BPD in the investigator’s judgment. Key exclusion criteria were inpatient status; concurrent DSM-5 diagnosis of schizophrenia or schizoaffective disorder; concurrent diagnosis of bipolar I or II disorder, delirium, dementia or other cognitive disorder, amnesia, eating disorder, antisocial personality disorder, or substance or alcohol use disorder; received psychotherapy for BPD symptoms (e.g., dialectical behaviour therapy or mentalisation-based therapy) within 60 days (individual/group supportive talk therapy and other non-pharmacological interventions may be permitted provided the treatment has not been initiated or changed within 60 days); a significant risk of committing violent acts, serious self-harm, or suicide based on history or routine psychiatric status examination (non-suicidal self-injurious behaviour and active suicidal ideation without specific plan were permitted); history of neuroleptic malignant syndrome, serotonin syndrome or clinically significant tardive dyskinesia; or prior exposure to brexpiprazole. Concurrent MDD, post-traumatic stress disorder, attention deficit hyperactivity disorder, panic disorder and generalised anxiety disorder were permitted provided that symptoms were stable and not the primary focus of treatment. Antipsychotics (other than the study drug), mood stabilisers and anticonvulsants were prohibited during the study, whereas benzodiazepines, hypnotics and antidepressants were permitted provided their use was chronic and stable.

The study comprised a 1-week placebo run-in followed by an 11-week randomised treatment phase in which patients were randomised 1:1 to oral brexpiprazole 2–3 mg/day once-daily (flexible dose) or placebo. To reduce potential bias, the existence of the placebo run-in and the timing of randomisation were blinded to investigators and patients (as was the timing of the primary and key secondary endpoints—see ‘Statistical analysis’ section). Brexpiprazole was titrated as follows: first week, 1 mg/day; second week, 2 mg/day; third week, 3 mg/day; thereafter, 2–3 mg/day. Outpatient visits occurred at screening, baseline (Day 0), randomisation (Week 1), and then every 2 weeks (Weeks 2–12), with a safety follow-up at Week 15 for patients who did not roll over into the extension study.

Randomisation was stratified by site, antidepressant treatment status (with/without) and symptom improvement during the placebo run-in (eligible/ineligible for enriched efficacy sample—see ‘Statistical analysis’ section). Brexpiprazole tablets and matching placebo were provided by the sponsor, packaged in numbered, weekly blister cards and assigned to patients using an ePlatform via a computer-generated randomisation code provided by the sponsor. Treatment assignments were blinded to patients, investigators and sponsor personnel, including those involved in data analysis.

#### Open-label extension study

This was a multicentre, open-label extension study of brexpiprazole in adult patients with BPD conducted at 55 sites in the United States and Europe (Spain, Ukraine) (ClinicalTrials.gov identifier: NCT04186403). Patients who completed the randomised controlled ‘parent’ study were eligible for enrolment.

The extension study comprised a 12-week treatment phase in which all patients received oral brexpiprazole 2–3 mg/day once-daily (flexible dose). Brexpiprazole was titrated for all patients, regardless of treatment received in the parent study (first week, 1 mg/day; second week, 2 mg/day; thereafter, 2–3 mg/day). Prohibited medications were the same as for the parent study; however, antidepressant dose changes were permitted, and other exceptions could be considered. Outpatient visits occurred at baseline (last visit of the parent study) and Weeks 2, 4, 8 and 12, with telephone contact at Weeks 1, 6 and 10, and a safety follow-up at Week 15.

### Assessments

Demographic information and medical history were recorded at screening. Sex at birth, race and ethnicity used U.S. Census Bureau classifications; the protocol did not specify a method of collection.

Efficacy was primarily assessed using the ZAN-BPD—a clinician-administered interview to assess the severity of the nine DSM diagnostic criteria for BPD; total scores range from 0 (best) to 36 (worst) (Zanarini, 2003). Efficacy was also assessed using the clinician-rated Clinical Global Impression – Severity of illness (CGI-S) and Improvement (CGI-I) scales (Guy, 1976), and patient-rated Patient’s Global Impression of Severity (PGI-S) and Change (PGI-C) scales; all are single items scored from 1 (best) to 7 (worst). Efficacy was assessed at each outpatient visit.

Safety was assessed by treatment-emergent adverse events (TEAEs, using Medical Dictionary for Regulatory Activities [MedDRA] terms), body weight, laboratory tests, vital signs, electrocardiograms (ECGs), the Columbia Suicide Severity Rating Scale (C-SSRS) (Posner *et al*., 2011) and three extrapyramidal symptom (EPS) rating scales: Simpson–Angus Scale (SAS) (Simpson and Angus, 1970), Abnormal Involuntary Movement Scale (AIMS) (Guy, 1976), and Barnes Akathisia Rating Scale (BARS) (Barnes, 1989).

### Statistical analysis

#### Randomised controlled study

The primary estimand was defined by the following components. Population: enriched efficacy sample (defined below). Treatments: brexpiprazole 2–3 mg/day or placebo. Endpoint: change from randomisation (Week 1) to Week 10 in ZAN-BPD total score. Measure of intervention effect: difference in endpoint means between brexpiprazole and placebo arms. Intercurrent events: premature treatment discontinuation. Hypothetical strategy: no occurrence of intercurrent events in the 12-week treatment period.

Efficacy was evaluated in an enriched sample that excluded patients who responded during the placebo run-in phase (and therefore may not benefit from randomised study drug). The enriched efficacy sample was defined as patients who received at least one post-randomisation dose of double-blind treatment, who had a randomisation (Week 1) and at least one post-randomisation ZAN-BPD total score rating, and whose symptoms did not improve above the following severity threshold during the placebo run-in: ZAN-BPD total score ≥10 and a score ≥2 on two or more specified items (inappropriate anger, paranoid ideation, affective instability, impulsivity) at randomisation (Week 1). Efficacy was also analysed in the full efficacy sample (i.e. regardless of whether or not patients met the enrichment criteria), and in a *post hoc* sample of patients who did not meet the enrichment criteria.

To reduce potential bias, the timing of the primary endpoint and the key secondary endpoint (change in CGI-S score from randomisation to Week 10) was blinded to investigators and patients. Other secondary endpoints were change in PGI-S score, and absolute CGI-I and PGI-C scores.

ZAN-BPD, CGI-S and PGI-S scores were analysed using a mixed model for repeated measures based on all observed-case data with fixed effects of treatment, site, visit, antidepressant treatment status (with/without), treatment-by-visit interaction, sex-by-visit interaction, and covariates of baseline-by-visit interaction and age-by-visit interaction. Under the hypothetical strategy, the event of withdrawing study drug was considered missing at random. CGI-I and PGI-C were analysed using last observation carried forward with Cochran–Mantel–Haenszel (CMH) row mean scores statistics controlling for site. Prespecified subgroup analyses of the primary endpoint were performed by sex (female, male), race (White, all other races), age (<55 years, ≥55 years), region (USA, Europe), and concomitant antidepressant use (yes, no).

A total of 200 patients (100 per arm) was projected to yield 80% power to detect a mean (standard deviation [SD]) between-group difference of −2.6 (6.5) points in the primary endpoint at a two-tailed significance level of 0.05. Assuming a 5% dropout rate and considering the enrichment criterion, 240 patients were planned for randomisation. To control for family-wise type I error, a testing hierarchy was employed in which subsequent statistical tests were performed only when all preceding *p*-values were significant: 1) primary endpoint in enriched efficacy sample; 2) key secondary endpoint in enriched efficacy sample; 3) primary endpoint in efficacy sample; 4) key secondary endpoint in efficacy sample.

The safety sample was defined as all patients who received at least one post-randomisation dose of double-blind treatment. Weight change was analysed using an analysis of covariance (ANCOVA) model with treatment as main effect and baseline as covariate. The incidence of ≥7% increase and decrease in body weight was compared using the CMH general association test. SAS, AIMS and BARS scores were analysed using ANCOVA with treatment and site as main effects and baseline value as covariate.

#### Open-label extension study

The primary endpoint was the frequency and severity of TEAEs over 12 weeks. Other safety analyses were secondary endpoints, and efficacy was assessed as an exploratory endpoint.

Sample size was based on the number of patients who rolled over from the parent study, not statistical power considerations. The safety sample was defined as all patients who received at least one dose of study drug. Efficacy was assessed in the efficacy sample, defined as patients in the safety sample who had at least one post-baseline ZAN-BPD total score rating.

Data were summarised using descriptive statistics for the total sample, and also by subgroup according to treatment received in the parent study (i.e. with/without previous exposure to brexpiprazole).

In both studies, analyses were performed using SAS version 9.4 (SAS Institute Inc; Cary, NC).

## Results

### Patients

#### Randomised controlled study

**Figure 1.**
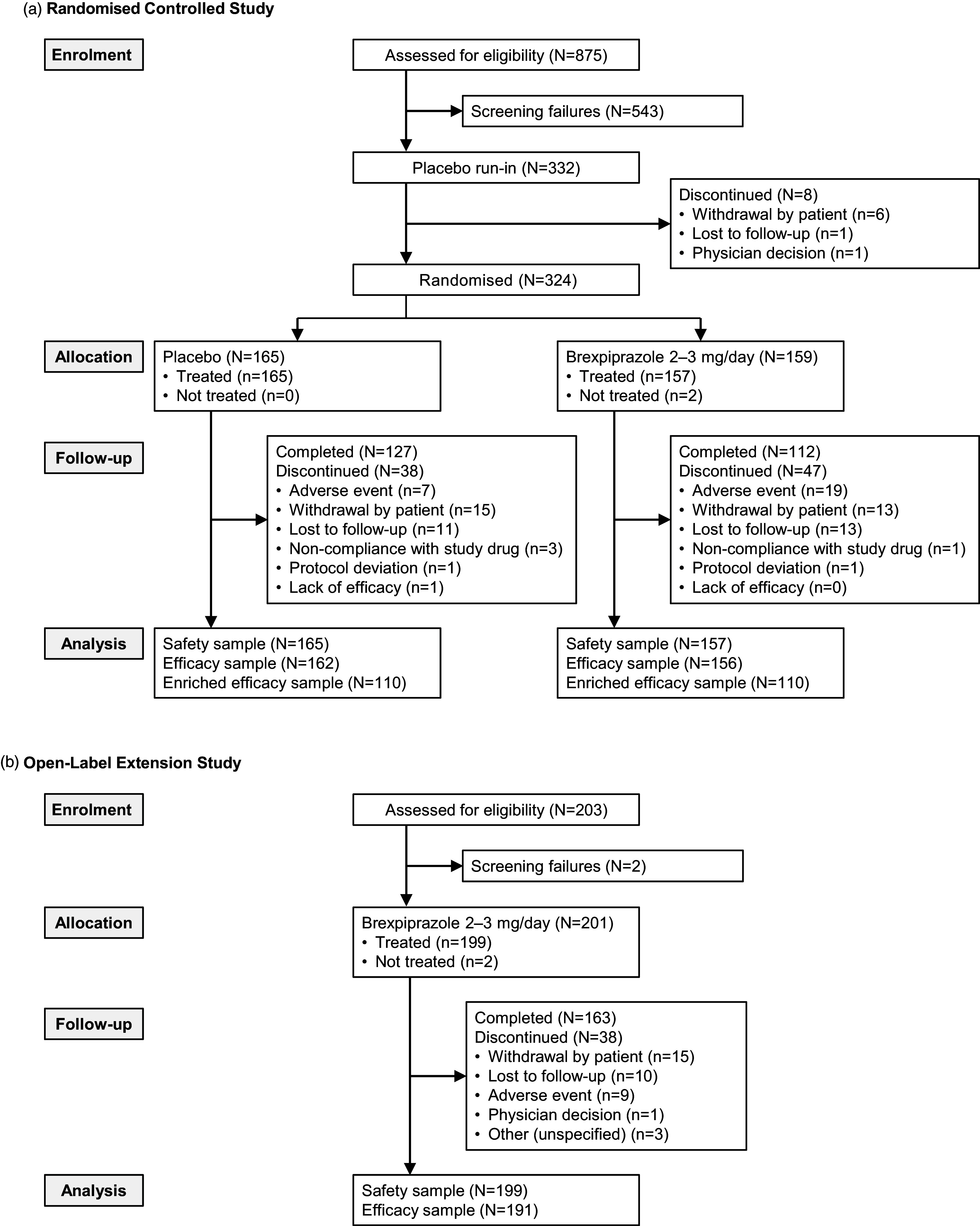
Study flow in A. the randomised controlled study and B. the open-label extension study.

The first patient was enrolled on 17 October 2019, and the last patient’s last visit was on 27 June 2021. Most patients were enrolled in the USA (92.9% of randomised sample). Completion rates were 70.4% for brexpiprazole and 77.0% for placebo; the most common reason for discontinuation of brexpiprazole was adverse events (11.9%, vs. 4.2% with placebo) (Figure 1A).

Baseline demographic and clinical characteristics were similar between treatment groups and indicated moderate severity of BPD (Table 1A). The majority of patients were female (82.1%) and White (78.4%). The most common psychiatric histories (>10%, MedDRA terms) were major depression (37.3%) and insomnia (11.4%).

**Table 1.** Demographic and clinical characteristics in A. the randomised controlled study and B. the open-label extension study

Time point/characteristic	A. Randomised controlled study								B. Open-label extension study					
	Randomised sample				Enriched efficacy sample				Enrolled sample					
	Placebo (*N* = 165)		Brexpiprazole (*N* = 159)		Placebo (*N* = 110)		Brexpiprazole (*N* = 110)		Brexpiprazole: total (*N* = 201)		Brexpiprazole: prior placebo subgroup (*n* = 111)		Brexpiprazole: prior brexpiprazole subgroup (*n* = 90)	
Screening	Mean	SD	Mean	SD	Mean	SD	Mean	SD	Mean	SD	Mean	SD	Mean	SD
Age (years)	31.0	10.9	32.0	10.6	31.1	11.3	31.4	9.7	32.7	10.6	32.0	10.6	33.6	10.5
Weight (kg)	78.4	22.3	78.6	22.6	77.5	21.7	76.0	19.9	80.6^a^	22.7	81.3^b^	23.4	79.7	21.9
BMI (kg/m^2^)	28.5	7.7	28.6	7.6	28.1	7.3	28.1	7.0	29.1^a^	7.9	29.6^b^	8.5	28.7	7.1
Time since initial BPD diagnosis (months)	42.0	82.8	49.3	69.9	34.8	74.1	43.2	65.5	43.4	76.5	36.9	76.5	51.3	76.3
Screening	*n*	%	*n*	%	*n*	%	*n*	%	*n*	%	*n*	%	*n*	%
Sex														
Female	137	83.0	129	81.1	91	82.7	95	86.4	163	81.1	92	82.9	71	78.9
Male	28	17.0	30	18.9	19	17.3	15	13.6	38	18.9	19	17.1	19	21.1
Race														
American Indian or Alaska Native	0	0.0	3	1.9	0	0.0	3	2.7	2	1.0	0	0.0	2	2.2
Asian	2	1.2	7	4.4	0	0.0	3	2.7	4	2.0	0	0.0	4	4.4
Black or African American	22	13.3	19	11.9	14	12.7	10	9.1	26	12.9	19	17.1	7	7.8
Native Hawaiian or Pacific Islander	1	0.6	1	0.6	0	0.0	1	0.9	1	0.5	1	0.9	0	0.0
White	131	79.4	123	77.4	91	82.7	91	82.7	161	80.1	89	80.2	72	80.0
Other	9	5.5	6	3.8	5	4.5	2	1.8	7	3.5	2	1.8	5	5.6
Ethnicity														
Hispanic or Latino	33	20.0	31	19.5	22	20.0	17	15.5	32	15.9	17	15.3	15	16.7
Not Hispanic or Latino	131	79.4	127	79.9	87	79.1	92	83.6	168	83.6	93	83.8	75	83.3
Unknown	0	0.0	1	0.6	0	0.0	1	0.9	0	0.0	0	0.0	0	0.0
Other	1	0.6	0	0.0	1	0.9	0	0.0	1	0.5	1	0.9	0	0.0
Antidepressant use	51	30.9	45	28.3	35	31.8	30	27.3	58	28.9	38	34.2	20	22.2
Baseline (Day 0)	Mean	SD	Mean	SD	Mean	SD	Mean	SD	Mean	SD	Mean	SD	Mean	SD
ZAN-BPD total score	18.2	4.0	18.8	4.0	19.0	4.1	19.6	4.1	9.0^c^	6.3	9.7	6.6	8.2^d^	5.8
CGI-S score	4.5	0.6	4.6	0.6	4.6	0.7	4.7	0.6	3.0^c^	1.2	3.2	1.2	2.9^d^	1.2
PGI-S score	4.6^e^	1.1	4.6^f^	1.1	4.7^g^	1.0	4.7^h^	1.0	3.4^i^	1.2	3.5^j^	1.3	3.3^k^	1.2
Randomisation (Week 1)	Mean	SD	Mean	SD	Mean	SD	Mean	SD	Mean	SD	Mean	SD	Mean	SD
ZAN-BPD total score	13.8	5.8	14.4	6.0	16.7	4.5	17.3	4.3	N/A	N/A	N/A	N/A	N/A	N/A
CGI-S score	3.9	0.9	4.0	1.0	4.3	0.8	4.4	0.7	N/A	N/A	N/A	N/A	N/A	N/A
PGI-S score	4.2^l^	1.1	4.1^m^	1.2	4.5^j^	1.0	4.4^j^	1.1	N/A	N/A	N/A	N/A	N/A	N/A

BMI, body mass index; BPD, borderline personality disorder; CGI-S, Clinical Global Impression – Severity of illness; N/A, not applicable; PGI-S, Patient’s Global Impression of Severity; SD, standard deviation; ZAN-BPD, Zanarini Rating Scale for BPD.

a *n* = 200.

b *n* = 110.

c *n* = 199.

d *n* = 88.

e *n* = 160.

f *n* = 156.

g *n* = 106.

h *n* = 107.

i *n* = 195.

j *n* = 108.

k *n* = 87.

l *n* = 163.

m *n* = 157.

At randomisation (Week 1), ZAN-BPD total scores were higher in the enriched efficacy sample (∼17 points) than in the randomised sample (Table 1A) and full efficacy sample (both ∼ 14 points).

The mean brexpiprazole dose at each patient’s last visit was 2.77 mg (*n* = 157). During the study, 38.8% of the safety sample took a concomitant psychiatric medication (placebo, 39.4%; brexpiprazole, 38.2%), most commonly (≥5%) fluoxetine (6.5%).

##### Open-label extension study

The first patient was enrolled on 13 January 2020, and the last patient’s last visit was on 22 September 2021. Most patients were enrolled in the USA (92.0%). Of 201 enrolled patients, 111 had received placebo during the parent study, and 90 had received brexpiprazole during the parent study. The completion rate was 81.1% (Figure 1B) and did not notably differ according to treatment received in the parent study (Table S1 in the online supplement).

Baseline demographic characteristics were representative of the parent study; baseline clinical characteristics indicated mild severity of BPD (Table 1B). The most common psychiatric histories (>10%, MedDRA terms) were major depression (37.8%), insomnia (17.9%), anxiety (10.4%) and depression (10.4%).

The mean brexpiprazole dose at each patient’s last visit was 2.32 mg in the total safety sample (prior placebo subgroup, 2.25 mg; prior brexpiprazole subgroup, 2.40 mg). During the study, 31.7% of patients took a concomitant psychiatric medication (34.2% in the prior placebo subgroup; 28.4% in the prior brexpiprazole subgroup), most commonly (≥5%) escitalopram (5.5%) and fluoxetine (5.0%).

### Efficacy

#### Randomised controlled study

**Figure 2.**
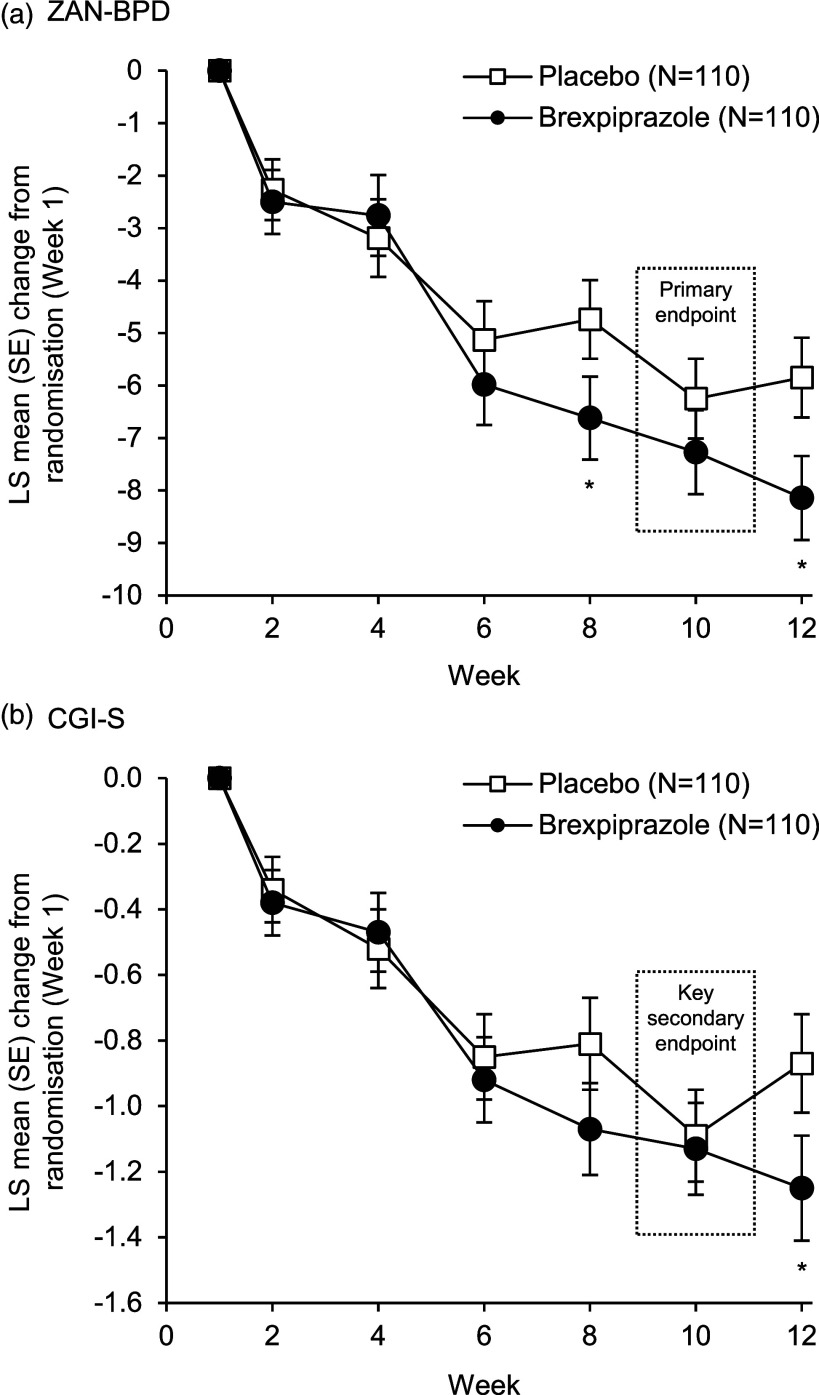
Mean change in A. ZAN-BPD total score and B. CGI-S score from randomisation (Week 1) in the randomised controlled study (enriched efficacy sample). CGI-S, Clinical Global Impression – Severity of illness; LS, least squares; SE, standard error; ZAN-BPD, Zanarini Rating Scale for Borderline Personality Disorder.*p < 0.05 versus placebo.

No statistically significant difference between brexpiprazole and placebo was observed on the primary endpoint analysis (enriched efficacy sample) of mean change in ZAN-BPD total score from randomisation (Week 1) to Week 10 (least squares mean difference [LSMD] −1.02; 95% confidence limits [CLs] −2.75, 0.70; test value −1.17; degrees of freedom 183; *p* = 0.24) (Figure 2A). Brexpiprazole was associated with nominally significant improvements in ZAN-BPD total score versus placebo at Week 8 (LSMD −1.88; 95% CLs −3.57, −0.19; *p* = 0.029) and Week 12 (LSMD −2.30; 95% CLs −4.04, −0.55; *p* = 0.010). On the key secondary endpoint analysis (enriched efficacy sample), mean change in CGI-S score from randomisation (Week 1) to Week 10 did not differ between brexpiprazole and placebo (LSMD −0.04; 95% CLs −0.35, 0.27; test value −0.29; degrees of freedom 186; *p* = 0.78); nominally significant separation was observed at Week 12 (LSMD −0.38; 95% CLs −0.72, −0.03; *p* = 0.031) (Figure 2B). There were no meaningful differences between groups on other secondary efficacy endpoints at Week 10 (Table S2 in the online supplement).

Subgroup analyses by sex, race, age, region, and concomitant antidepressant use generally aligned with the results of the primary analysis (Fig. S1 in the online supplement). Subgroups with ≤ 10 patients per treatment arm (e.g. Europe) were too small to allow definitive conclusions.

Mean changes in ZAN-BPD total score and CGI-S score from randomisation (Week 1) to Week 10, and treatment differences at Week 10, were smaller in the full efficacy sample than in the enriched efficacy sample (Fig. S2 in the online supplement). Mean change in ZAN-BPD total score was minimal in patients who did not meet the criteria for enrichment (*post hoc* analysis) (Fig. S3 in the online supplement).

#### Open-label extension study

Mean (SD) ZAN-BPD total score improved from baseline to last visit in the total efficacy sample (−2.8 [5.2]; *N* = 191). The improvement was numerically greater in the subgroup of patients who received placebo during the parent study (−3.7 [5.3]; *n* = 107) than in the subgroup who previously received brexpiprazole (−1.7 [4.9]; *n* = 84). Similarly, mean (SD) CGI-S score improved from baseline to last visit in the total sample (−0.6 [1.1]; *N* = 191), and improvement was numerically greater in the subgroup who had previously received placebo (−0.8 [1.1]; *n* = 107) compared with the subgroup who previously received brexpiprazole (−0.4 [1.0]; *n* = 84).

### Safety

#### Randomised controlled study

The overall incidence of TEAEs was 60.5% with brexpiprazole and 47.9% with placebo. TEAEs with incidence ≥5% for brexpiprazole and greater incidence than placebo were akathisia, insomnia, anxiety, fatigue, weight increased, restlessness, somnolence and increased appetite (Table 2A). Most TEAEs were mild or moderate in severity, and there were no deaths during the study.

**Table 2.** Summary of TEAEs in A. the randomised treatment phase of the randomised controlled study and B. the open-label extension study (safety sample)

TEAE	A. Randomised controlled study				B. Open-label extension study					
	Placebo (*N* = 165)		Brexpiprazole (*N* = 157)		Brexpiprazole: total (*N* = 199)		Brexpiprazole: prior placebo subgroup (*n* = 111)		Brexpiprazole: prior brexpiprazole subgroup (*n* = 88)	
	*n*	%	*n*	%	*n*	%	*n*	%	*n*	%
At least one TEAE	79	47.9	95	60.5	86	43.2	56	50.5	30	34.1
At least one serious TEAE	2	1.2^a^	5	3.2^b^	3	1.5^c^	2	1.8	1	1.1
Discontinuation due to AEs	6	3.6^d^	18	11.5^e^	9	4.5^f^	8	7.2	1	1.1
TEAEs with incidence ≥5% for brexpiprazole and >placebo in the randomised controlled study^g^										
Akathisia	2	1.2	22	14.0	10	5.0	9	8.1	1	1.1
Insomnia	10	6.1	15	9.6	11	5.5	6	5.4	5	5.7
Anxiety	9	5.5	13	8.3	6	3.0	3	2.7	3	3.4
Fatigue	6	3.6	12	7.6	8	4.0	7	6.3	1	1.1
Weight increased	4	2.4	10	6.4	6	3.0	6	5.4	0	0.0
Restlessness	2	1.2	10	6.4	8	4.0	7	6.3	1	1.1
Somnolence	5	3.0	8	5.1	3	1.5	3	2.7	0	0.0
Increased appetite	4	2.4	8	5.1	1	0.5	0	0.0	1	1.1

AE, adverse event; TEAE, treatment-emergent adverse event.

a Dissociation, gastritis, pneumonia (some patients reported >1).

b Suicide attempt (2), cerebrovascular accident, major depression, panic attack.

c Suicidal ideation (2), contusion.

d Confusional state, dissociation, disturbance in attention, insomnia, lethargy, migraine, somnolence (some patients reported >1).

e Akathisia (4), depression (3), anxiety (2), major depression (2), tremor (2), agitation, cerebrovascular accident, disorientation, fatigue, increased appetite, increased weight, muscle spasms, panic attack, restlessness, suicidal ideation, suicide attempt (some patients reported >1).

f Akathisia (2), suicidal ideation (2), diarrhoea, disturbance in attention, feeling abnormal, increased weight, nausea, panic attack, restlessness, tongue disorder (some patients reported >1).

g And their corresponding incidences in the open-label extension study.

Mean weight change from randomisation (Week 1) to last visit was greater for brexpiprazole than placebo (LSMD 1.47 kg; 95% CLs 0.84, 2.09; *p* < 0.001) (Table S3A in the online supplement). Mean changes in laboratory test parameters were generally small and similar between treatment groups; the exception was triglycerides, which increased in the brexpiprazole group but not in the placebo group (Table S3A in the online supplement). Mean changes in vital signs and ECG parameters were similar between treatment groups (data not shown). There were two events of potentially clinically relevant ECG abnormality in each treatment group.

According to the C-SSRS, 21.7% (34/157) of patients in the brexpiprazole group and 23.6% (39/165) in the placebo group had findings suggestive of emergence of any type of suicidal ideation at any post-randomisation visit, and 2.5% (4/157) and 1.2% (2/165), respectively, had emergence of any type of suicidal behaviour.

Mean (SD) changes in EPS scale scores from baseline to last visit were minimal (≤0.1 for brexpiprazole and placebo). The incidence of moderate-to-severe akathisia (BARS global score of 3–5) at any visit was 2.5% (4/157) for brexpiprazole and 0.0% (0/165) for placebo. The incidence of EPS-related TEAEs was 16.6% (26/157) for brexpiprazole and 1.8% (3/165) for placebo.

#### Open-label extension study

The incidence of TEAEs was 43.2% overall, and numerically lower among patients who had received brexpiprazole during the parent study (34.1%) compared with patients who had received placebo (50.5%). TEAEs with incidence ≥5% were insomnia and akathisia (Table 2B). Most TEAEs were mild or moderate in severity, and there were no deaths during the study.

Mean weight change was numerically smaller among patients who had received brexpiprazole during the parent study compared with patients who had received placebo (Table S3B in the online supplement). Mean changes in laboratory test parameters were generally small; triglycerides increased by a numerically greater amount in the subgroup who had received placebo in the parent study than in the subgroup who had received brexpiprazole (Table S3B in the online supplement). Mean changes in vital signs and ECG parameters were similar between the prior brexpiprazole and prior placebo subgroups (data not shown). There were four events of potentially clinically relevant ECG abnormality, all in patients who had received placebo in the parent study.

According to the C-SSRS, 13.6% (27/199) of patients had findings suggestive of emergence of any type of suicidal ideation during the study, and 1.0% (2/199) had emergence of any type of suicidal behaviour, with no notable differences between the prior brexpiprazole and prior placebo subgroups.

Mean changes from baseline in EPS scale scores were 0.0 for all scales in the total safety sample, with no notable differences between the prior brexpiprazole and prior placebo subgroups. The incidence of moderate-to-severe akathisia (BARS global score of 3–5) at any visit was 2.5% (5/199); one of these patients had previous exposure to brexpiprazole in the parent study. The incidence of EPS-related TEAEs was 7.0% (14/199): 10.8% (12/111) in the prior placebo subgroup and 2.3% (2/88) in the prior brexpiprazole subgroup.

## Discussion

In the 12-week randomised controlled study, brexpiprazole was not statistically significantly different from placebo on the primary efficacy endpoint (ZAN-BPD) or the key secondary efficacy endpoint (CGI-S) at Week 10. The primary and key secondary efficacy endpoints were analysed at Week 10 rather than Week 12 (a fact that was blinded to investigators and patients) to reduce the bias that may arise from knowing the timing of endpoints. Brexpiprazole did show greater improvement than placebo in exploratory analyses of the primary efficacy endpoint at Weeks 8 and 12 (which were outside the formal testing hierarchy and should therefore be interpreted with caution). It is unclear why brexpiprazole showed improvements versus placebo before and after Week 10, but not at Week 10, and further research is needed. Of note, ZAN-BPD total score consistently continued to improve over Weeks 6–12 in the brexpiprazole group, whereas improvement in the placebo group fluctuated from week to week (Figure 2A).

In a prior randomised controlled study in BPD, brexpiprazole showed greater improvement versus placebo on ZAN-BPD total score at Week 12, though not at earlier time points (Grant *et al*., 2021). The lack of effect at earlier time points was partly attributed to a robust placebo response. To reduce the placebo effect in the present randomised study, a double-blind placebo run-in was used to enrich the sample for patients who continued to meet minimum severity criteria after 1 week on placebo. This enrichment strategy enhanced the ZAN-BPD drug–placebo difference in the randomised treatment phase. The drug–placebo difference was minimal in the subgroup who did not meet the criteria for enrichment; this may be a flooring effect due to large improvements during the placebo run-in.

In the open-label extension study, ZAN-BPD total score improved by a greater amount in the subgroup without (versus with) previous exposure to brexpiprazole. This suggests that most of the improvement associated with brexpiprazole occurred in the first 12 weeks of treatment, which was then maintained beyond 12 weeks.

Considering other atypical antipsychotics, a systematic review of randomised controlled trials concluded that olanzapine and quetiapine had no effect on reducing the severity of BPD as measured by ZAN-BPD total score, with low certainty of evidence (Schulz *et al*., 2008; Zanarini *et al*., 2011; Black *et al*., 2014; Gartlehner *et al*., 2021). In general, clinical trials in BPD are challenging due to the nature of the population (i.e. symptom heterogeneity, high rates of comorbidity, and concomitant psychiatric medication) (Zanarini *et al*., 2010), issues with participant recruitment and retention (Woo *et al*., 2021), and a large placebo response (Zanarini *et al*., 2011; Black *et al*., 2014; Grant *et al*., 2021). In other psychiatric disorders, meta-analyses of randomised clinical trials show that atypical antipsychotics, including brexpiprazole, have generally similar efficacy on overall symptoms of schizophrenia and for the adjunctive treatment of MDD, but differ in terms of their safety profiles (Citrome, 2017; Huhn *et al*., 2019; Schneider-Thoma *et al*., 2022; Kishimoto *et al*., 2023).

Brexpiprazole had a similar safety profile in BPD to that established in schizophrenia and MDD (Marder *et al*., 2017; Thase *et al*., 2019). The incidence of akathisia with brexpiprazole was 14.0% in the randomised study, but akathisia generally did not result in treatment discontinuation. Only 1.1% of patients with previous exposure to brexpiprazole had akathisia in the extension study, suggesting that this event may stabilise after initial treatment. Mean weight gain also appeared to stabilise in the extension study among patients with previous exposure to brexpiprazole. High treatment discontinuation rates are well documented in BPD trials, attributed to dissatisfaction with treatment and the nature of the disorder, among other reasons (Iliakis *et al*., 2021). Treatment discontinuation rates in the present trials were similar to those reported in prior randomised trials of atypical antipsychotics in BPD (Schulz *et al*., 2008; Zanarini *et al*., 2011; Black *et al*., 2014).

The present studies are limited in that the patient sample was approximately 80% White, meaning that findings may not generalise to non-White participants. Over 90% of the study sample was from the USA; the inclusion of a small number of patients from Europe increased generalizability but may have introduced bias. However, subgroup analyses indicated that results in the USA were aligned with the overall analysis, as would be expected. As with all clinical trials, patient selection criteria and restrictions (e.g. the exclusion of adolescents) limit generalizability to a broader patient population. Patients who received psychotherapy for BPD symptoms were excluded to avoid confounding effects; however, this limits external validity, since psychotherapy is the mainstay of BPD treatment (Simonsen *et al*., 2019). Additionally, one of the inclusion criteria was that patients must require treatment with a medication for BPD in the investigator’s judgment; in the absence of approved medications for BPD, this was a subjective criterion to allow for investigator discretion in the suitability of patients for enrolment.

In conclusion, the blinded primary endpoint of the Phase 2 randomised study was not met. Numerical efficacy advantages were observed at certain time points, and further research is needed to determine the specific value of brexpiprazole in the difficult-to-treat population of patients with BPD. Adverse effects were mostly mild or moderate in severity, and were generally transient, based on data from the open-label study. Finally, the randomised study provided important insights regarding trial design: that enrichment of the efficacy sample enhanced the drug–placebo difference, whereas blinding of the primary endpoint did not.

## Supporting information

Rothman et al. supplementary material Rothman et al. supplementary material

## Supplementary material

The supplementary material for this article can be found at https://doi.org/10.1017/neu.2024.31.
