# Supplementary material for: A randomised study and an extension study of brexpiprazole in patients with borderline personality disorder
Source: Acta Neuropsychiatr. 2024 Nov 19;37:e39. doi: 10.1017/neu.2024.31 (PMC13130291; doi:10.1017/neu.2024.31)
Supplement: Rothman et al. supplementary material [file S0924270824000310sup001.pdf]

Supplementary material

Supplement to: A randomized study and an extension study of brexpiprazole in patients with borderline personality disorder

Brian Rothman, Claudette Brewer, Denise Chang, Mary Hobart, Nanco Hefting, Robert D. McQuade, Jon E. Grant

Contents

Table S1. Patient disposition in the open-label extension study stratified by treatment received in the parent study (enrolled sample) 2

Table S2. Summary of efficacy endpoints in the randomized controlled study (enriched efficacy sample) 3

Table S3. Laboratory assessments and body weight in A. the randomized controlled study and B. the open-label extension study (safety sample) 4

Table S4. List of independent ethics committees (IECs)/institutional review boards (IRBs) 6

Fig. S1. Mean change in ZAN-BPD total score from randomization (Week 1) to Week 10 in the randomized controlled study, by subgroup (enriched efficacy sample). 7

Fig. S2. Mean change in A. ZAN-BPD total score and B. CGI-S score from randomization (Week 1) in the randomized controlled study (full efficacy sample[a]). 8

Fig. S3. Mean change in ZAN-BPD total score from randomization (Week 1) in the randomized controlled study, by enrichment status[a]. 9

Table S1. Patient disposition in the open-label extension study stratified by treatment received in the parent study (enrolled sample)

	Brexpiprazole: total (N=201)		Brexpiprazole: prior placebo subgroup (n=111)		Brexpiprazole: prior brexpiprazole subgroup (n=90)	
	n	%	n	%	n	%
Treated	199	99.0	111	100.0	88	97.8
Completed	163	81.1	92	82.9	71	78.9
Discontinued	38	18.9	19	17.1	19	21.1
Withdrawal by patient	15	7.5	8	7.2	7	7.8
Lost to follow-up	10	5.0	3	2.7	7	7.8
Adverse event	9	4.5	8	7.2	1	1.1
Physician decision	1	0.5	0	0.0	1	1.1
Other (unspecified)	3	1.5	0	0.0	3	3.3
Safety sample	199	99.0	111	100.0	88	97.8
Efficacy sample	191	95.0	107	96.4	84	93.3

Table S2. Summary of efficacy endpoints in the randomized controlled study (enriched efficacy sample)

Endpoint	Treatment group	N	Randomization (Week 1), mean (SD)	Change at Week 10, LS mean (SE)	Treatment difference at Week 10 versus placebo	
					LS mean difference (95% CLs)	p-value
ZAN-BPD total score (primary)[a]	Brexpiprazole	110	17.3 (4.3)	-7.3 (0.8)	-1.02 (-2.75, 0.70)	0.24
	Placebo	110	16.7 (4.5)	-6.3 (0.8)		
CGI-S score (key secondary)[a]	Brexpiprazole	110	4.4 (0.7)	-1.1 (0.1)	-0.04 (-0.35, 0.27)	0.78
	Placebo	110	4.3 (0.8)	-1.1 (0.1)		
CGI-I score[b]	Brexpiprazole	110	N/A	2.5 (1.2)[c]	-0.20 (-0.51, 0.11)[d]	0.21
	Placebo	110	N/A	2.6 (1.2)[c]		
PGI-S score[a]	Brexpiprazole	108	4.4 (1.1)	-0.9 (0.2)	-0.11 (-0.44, 0.22)	0.51
	Placebo	108	4.5 (1.0)	-0.8 (0.1)		
PGI-C score[b]	Brexpiprazole	109	N/A	2.9 (1.3)[c]	-0.08 (-0.40, 0.25)[d]	0.65
	Placebo	110	N/A	3.0 (1.2)[c]		

CGI-I, Clinical Global Impression – Improvement; CGI-S, Clinical Global Impression – Severity of illness; CL, confidence limit; LOCF, last observation carried forward; LS, least squares; MMRM, mixed model for repeated measures; N/A, not applicable; PGI-C, Patient's Global Impression of Change; PGI-S, Patient's Global Impression of Severity; SD, standard deviation; SE, standard error; ZAN-BPD, Zanarini Rating Scale for borderline personality disorder.

[a]MMRM.

[b]LOCF.

[c]Mean (SD) score at Week 10.

[d]Adjusted mean difference (95% CLs) based on the Cochran–Mantel–Haenszel row mean scores differ test.

Table S3. Laboratory assessments and body weight in A. the randomized controlled study and B. the open-label extension study (safety sample)

	A. Randomized controlled study						B. Open-label extension study								
Assessment	Placebo			Brexpiprazole			Brexpiprazole: total			Brexpiprazole: prior placebo subgroup			Brexpiprazole: prior brexpiprazole subgroup		
Fasting serum parameter	N[a]	Mean change[b]	SD	N[a]	Mean change[b]	SD	N[a]	Mean change[b]	SD	N[a]	Mean change[b]	SD	N[a]	Mean change[b]	SD
Glucose (mg/dL)	126	0.31	15.30	116	0.97	12.44	111	3.47	26.46	62	2.68	33.26	49	4.47	14.03
HbA1c (%)	143	0.02	0.28	130	-0.01	0.25	165	-0.01	0.32	93	-0.03	0.36	72	0.01	0.25
Total cholesterol (mg/dL)	120	0.30	24.51	112	1.72	26.77	111	-1.38	22.27	62	3.52	22.62	49	-7.57	20.40
HDL cholesterol (mg/dL)	120	-0.32	9.49	112	-1.19	9.30	109	0.17	7.96	61	1.64	7.99	48	-1.71	7.59
LDL cholesterol (mg/dL)	120	0.60	23.12	112	-0.38	27.08	109	-3.70	18.51	61	-1.15	18.79	48	-6.94	17.82
Triglycerides (mg/dL)	120	0.10	44.95	112	16.80	54.06	109	6.77	48.48	61	10.98	44.50	48	1.42	53.10
Prolactin (ng/mL)															
Female	117	-1.36	12.61	106	9.43	12.05	132	3.99	13.55	77	9.79	12.23	55	-4.14	10.92
Male	26	-0.89	5.60	24	3.45	5.08	33	1.27	5.08	16	3.15	5.14	17	-0.50	4.48
Weight parameter	N[a]	LS mean change[b]	SD	N[a]	LS mean change[b]	SD	N[a]	Mean change[b]	SD	N[a]	Mean change[b]	SD	N[a]	Mean change[b]	SD
Weight (kg)	165	0.17	2.66	154	1.63***	3.01	193	1.6	3.7	109	2.5	3.7	84	0.5	3.4
Fasting serum shifts	N[c]	n[d]	%	N[c]	n[d]	%	N[c]	n[d]	%	N[c]	n[d]	%	N[c]	n[d]	%
Glucose N/I→H	125	5	4.0	116	5	4.3	108	3	2.8	60	1	1.7	48	2	4.2
Glucose I→H	16	2	12.5	11	1	9.1	19	3	15.8	11	1	9.1	8	2	25.0
HDL cholesterol N→L	56	0	0.0	55	0	0.0	97	5	5.2	57	4	7.0	40	1	2.5
LDL cholesterol N/B→H	113	4	3.5	104	7	6.7	102	1	1.0	58	1	1.7	44	0	0.0
Triglycerides N→H	106	3	2.8	96	9	9.4	92	1	1.1	53	1	1.9	39	0	0.0
Triglycerides N/B→H	111	4	3.6	102	11	10.8	99	4	4.0	56	1	1.8	43	3	7.0

Prolactin shifts	N[e]	n[f]	%	N[e]	n[f]	%	N[e]	n[f]	%	N[e]	n[f]	%	N[e]	n[f]	%
Prolactin >2x ULN															
Female	117	1	0.9	106	1	0.9	136	2	1.5	79	2	2.5	57	0	0.0
Male	26	0	0.0	24	0	0.0	34	0	0.0	17	0	0.0	17	0	0.0
Prolactin >3x ULN															
Female	117	0	0.0	106	0	0.0	136	0	0.0	79	0	0.0	57	0	0.0
Male	26	0	0.0	24	0	0.0	34	1	2.9	17	0	0.0	17	1	5.9
Weight shifts	N[e]	n[d]	%	N[e]	n[d]	%	N[e]	n[d]	%	N[e]	n[d]	%	N[e]	n[d]	%
≥7% increase	165	12	7.3	154	25	16.2	194	35	18.0	110	26	23.6	84	9	10.7
≥7% decrease	165	3	1.8	154	5	3.2	194	11	5.7	110	4	3.6	84	7	8.3

B, borderline; H, high; HbA1c, glycated hemoglobin; HDL, high-density lipoprotein; I, impaired; L, low; LDL, low-density lipoprotein; LS, least squares; N, normal; SD, standard deviation; ULN, upper limit of normal.

Categorical shifts in serum parameters were as follows: glucose N/I→H (<126 mg/dL to ≥126 mg/dL); glucose I→H (≥100 and <126 mg/dL to ≥126 mg/dL); HDL cholesterol N→L (≥40 mg/dL to <40 mg/dL); LDL cholesterol N/B→H (<160 mg/dL to ≥160 mg/dL); triglycerides N→H (<150 mg/dL to ≥200 and <500 mg/dL); triglycerides N/B→H (<200 mg/dL to ≥200 and <500 mg/dL).

***p<0.001 versus placebo (tests only performed for mean weight change).

[a]Number of patients with a baseline and post-baseline measurement.

[b]Mean change from baseline to last available post-baseline evaluation.

[c]Number of patients in the category at baseline who had a post-baseline measurement.

[d]Number of patients with this categorical shift at any post-baseline visit.

[e]Number of patients with a post-baseline measurement.

[f]Number of patients meeting this criteria at any post-baseline visit who did not meet the criteria at baseline (patients were counted once in the highest category that applied). In the randomized controlled study, baseline measurements were either taken at screening or Day 0 (except weight, where changes from randomization [Week 1] are presented); in the open-label extension study, baseline measurements were taken from the last scheduled treatment visit of the previous double-blind study.

Table S4. List of independent ethics committees (IECs)/institutional review boards (IRBs)

IEC/IRB type and country	IEC/IRB name and location
Central IRB – US	Advarra Institutional Review Board (Columbia, MD)
Local IRB – US	The University of Chicago Hospitals Office of Clinical Research, Section of Regulatory Compliance (Chicago, IL)
Central IEC – Spain	CEIm Parc Salut Mar, Institut Hospital del Mar d'Investigacions Mèdiques Edificio Parc de Recerca Biomèdica de Barcelona (Barcelona)
Local IEC – Ukraine	Communal Noncommercial Enterprise "Vinnytsia Regional Clinical Psychoneurological Hospital named after Acad. O. I. Yuschenko of Vinnytsia Regional Council" (Vinnytsia)
Local IEC – Ukraine	State Institution "Institute of Neurology, Psychiatry and Narcology of the National Academy of Medical Sciences of Ukraine" (Kharkiv)
Local IEC – Ukraine	Public Non-Profit Enterprise "Odesa Regional Medical Center of Mental Health" of Odesa Regional Council (Odesa)
Local IEC – Ukraine	Kyiv Railway Clinical Hospital #1 of Branch "Health Center" of joint stock company "Ukrainian Railway" (Kyiv)
Local IEC – Ukraine	Communal Enterprise "Regional Institution of Mental Psychiatric Care of the Poltava Regional Council" (Poltava)

Fig. S1. Mean change in ZAN-BPD total score from randomization (Week 1) to Week 10 in the randomized controlled study, by subgroup (enriched efficacy sample).

Subgroup	Brex n=	Pbo n=	LS mean difference (95% CI)
**Sex**			
Female	95	91	-0.58 (-2.46 to 1.30)
Male	15	19	-2.32 (-7.54 to 2.89)
**Race**			
White	91	91	-0.63 (-2.51 to 1.24)
All other races	19	19	-0.88 (-5.62 to 3.86)
**Age**			
<55 years	108	102	-0.75 (-2.56 to 1.06)
≥55 years	2	8	2.79 (-0.16 to 5.74)
**Region**			
USA	100	103	-0.39 (-2.21 to 1.43)
Europe	10	7	-8.48 (-13.0 to -3.92)
**Concomitant antidepressant**			
Yes	30	35	-2.30 (-5.56 to 0.95)
No	80	75	-0.31 (-2.37 to 1.75)

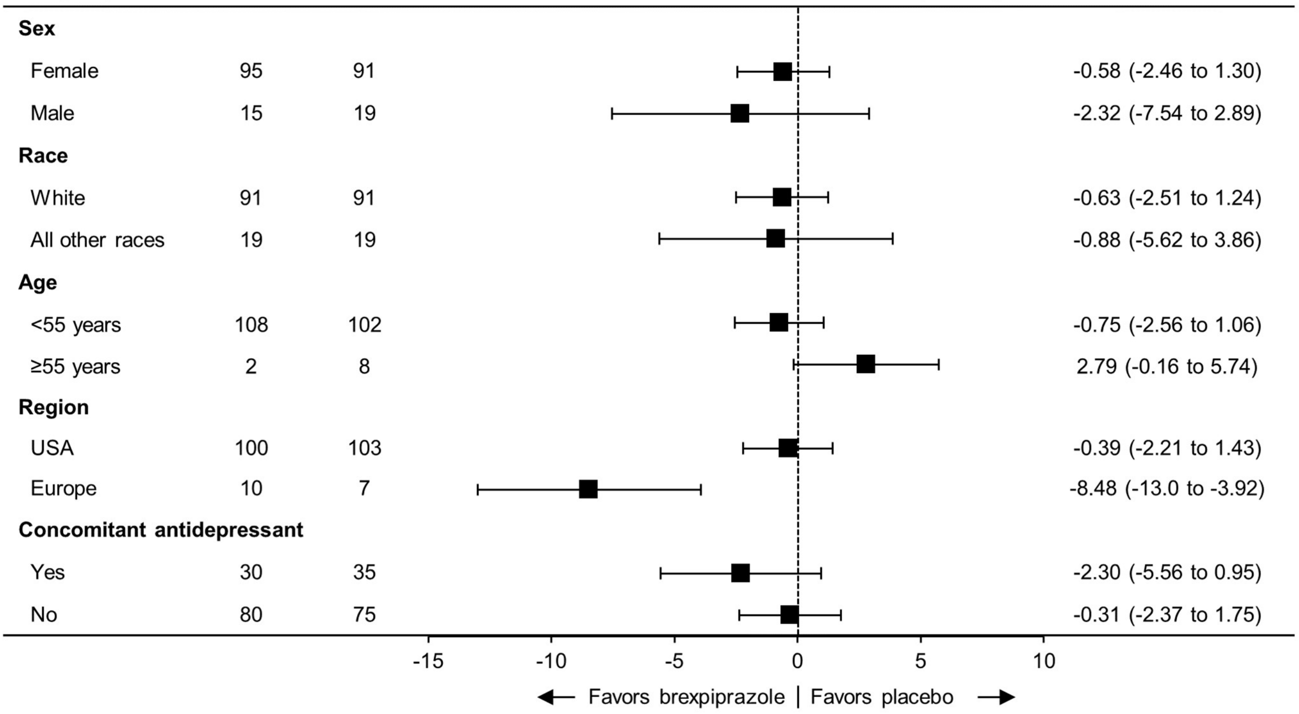

CI, confidence interval; LS, least squares; ZAN-BPD, Zanarini Rating Scale for Borderline Personality Disorder.

Fig. S2. Mean change in A. ZAN-BPD total score and B. CGI-S score from randomization (Week 1) in the randomized controlled study (full efficacy sample[a]).

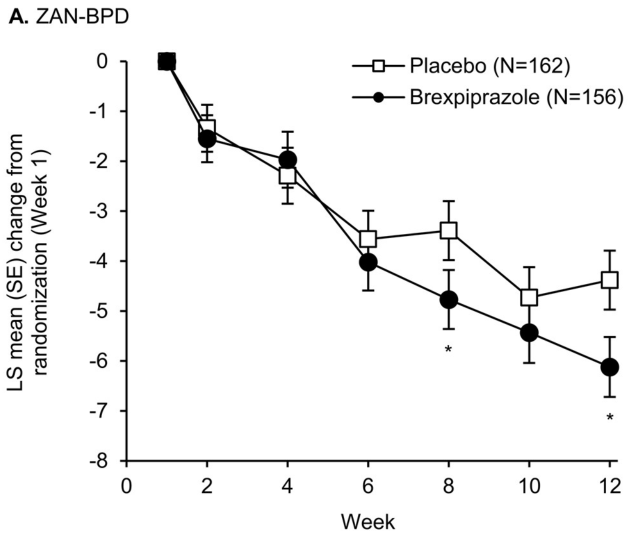

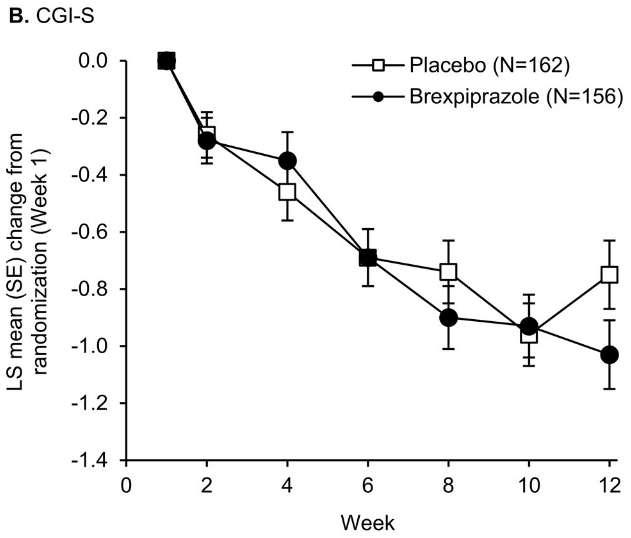

[a]To reduce the placebo effect, the primary analysis was performed in an enriched sample of patients who continued to meet minimum severity criteria after 1 week on placebo. The data presented here are for the full efficacy sample (patients who met the enrichment criteria plus those who did not). Mean score changes from randomization (Week 1) to Week 10, and treatment differences at Week 10, were smaller in the full efficacy sample than in the enriched efficacy sample, indicating the usefulness of enrichment to enhance the drug–placebo difference.

CGI-S, Clinical Global Impression – Severity of illness; LS, least squares; SE, standard error; ZAN-BPD, Zanarini Rating Scale for Borderline Personality Disorder.

*$p<0.05$ versus placebo.

Fig. S3. Mean change in ZAN-BPD total score from randomization (Week 1) in the randomized controlled study, by enrichment status[a].

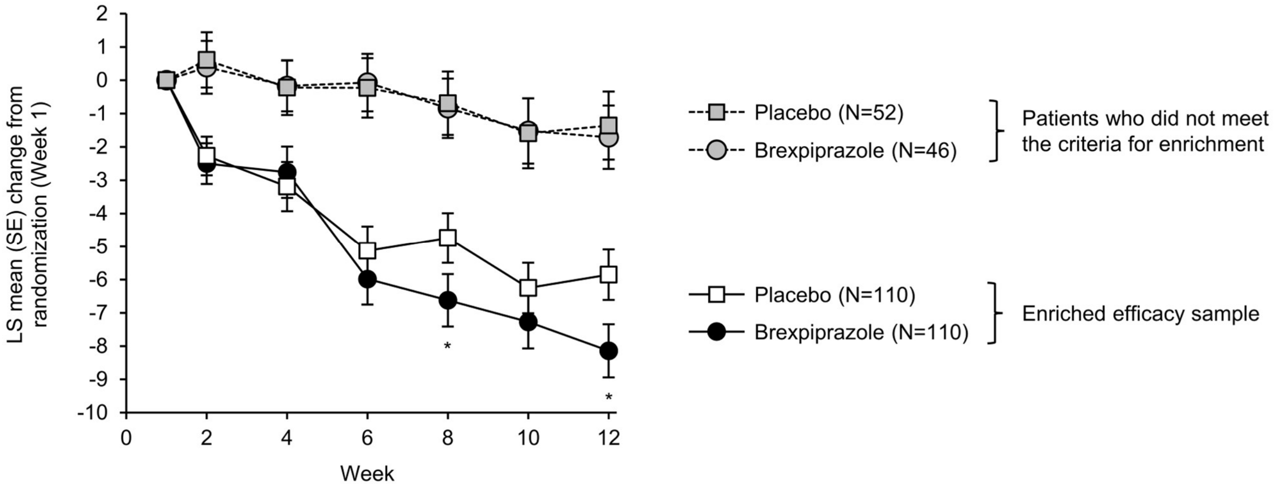

[a]To reduce the placebo effect, the primary analysis was performed in an enriched sample of patients who continued to meet minimum severity criteria after 1 week on placebo. The data presented here are stratified into subgroups for: 1) patients who met the enrichment criteria (data duplicated from Fig. 2); and 2) patients who did not meet the enrichment criteria. In the enriched efficacy sample (primary analysis), there was no statistically significant difference at Week 10, but possible efficacy signals at other time points. In patients who did not meet the criteria for enrichment (*post hoc* analysis), mean score change was minimal; this may be a flooring effect due to large improvements during the placebo run-in.

LS, least squares; SE, standard error; ZAN-BPD, Zanarini Rating Scale for Borderline Personality Disorder.

*$p<0.05$ versus placebo.
